# Influence of pregnancy and labor on the occurrence of nerve fibers expressing the capsaicin receptor TRPV1 in human corpus and cervix uteri

**DOI:** 10.1186/1477-7827-6-8

**Published:** 2008-02-12

**Authors:** Berith K Tingåker, Gunvor Ekman-Ordeberg, Paul Facer, Lars Irestedt, Praveen Anand

**Affiliations:** 1 Karolinska Institutet, Department of Woman and Child Health, Division of Obstetrics and Gynecology, Karolinska Hospital, SE-171 76 Stockholm, Sweden; 2 Karolinska Institutet, Department of Physiology and Pharmacology, Section of Anaesthesiology and Intensive Care, Karolinska University Hospital, Solna, SE-171 76 Stockholm, Sweden; 3 Peripheral Neuropathy Unit, Hammersmith Hospital, Faculty of Medicine, Imperial College London, London, UK

## Abstract

**Background:**

Cervical ripening is a prerequisite for a normal obstetrical outcome. This process, including labor, is a painful event that shares features with inflammatory reactions where peripheral nociceptive pathways are involved. The capsaicin and heat receptor TRPV1 is a key molecule in sensory nerves involved in peripheral nociception, but little is known regarding its role in the pregnant uterus. Therefore, the aim of this study was to investigate human corpus and cervix uteri during pregnancy and labor and non-pregnant controls for the presence of TRPV1.

**Methods:**

We have investigated human uterine corpus and cervix biopsies at term pregnancy and parturition. Biopsies were taken from the upper edge of the hysterotomy during caesarean section at term (n = 8), in labor (n = 8) and from the corresponding area in the non-pregnant uterus after hysterectomy (n = 8). Cervical biopsies were obtained transvaginally from the anterior cervical lip. Serial frozen sections were examined immunohistochemically using specific antibodies to TRPV1 and nerve markers (neurofilaments/peripherin).

**Results:**

In cervix uteri, TRPV1-immunoreactive fibers were scattered throughout the stroma and around blood vessels, and appeared more frequent in the sub-epithelium. Counts of TRPV1-immunoreactive nerve fibers were not significantly different between the three groups. In contrast, few TRPV1-immunoreactive fibers were found in nerve fascicles in the non-pregnant corpus, and none in the pregnant corpus.

**Conclusion:**

In this study, TRPV1 innervation in human uterus during pregnancy and labor is shown for the first time. During pregnancy and labor there was an almost complete disappearance of TRPV1 positive nerve fibers in the corpus. However, cervical innervation remained throughout pregnancy and labor. The difference in TRPV1 innervation between the corpus and the cervix is thus very marked. Our data suggest that TRPV1 may be involved in pain mechanisms associated with cervical ripening and labor. Furthermore, these data support the concept that cervix uteri may be the major site from which labor pain emanates. Our findings also support the possibility of developing alternative approaches to treat labor pain.

## Background

Giving birth is often very painful. Consequently, there is a demand for easily accessible and effective relief of labor pain. Various forms of central neuroaxial blockades (CNB) are currently the most efficient methods to alleviate labor pain and widely used in the "Developed world"[1]. However, from a global point of view, very few women have access to efficacious labor pain relief. This reality challenged us to study nerve-related changes taking place in corpus and cervix uteri during pregnancy and labor in order to increase our understanding of mechanisms related to labor pain.

The changes taking place in the extracellular matrix (ECM) of human corpus and cervix uteri related to the onset and progress of labor and parturition have been a focus of interest to our group for many years [2,3]. Anatomically, corpus uteri and cervix constitute one organ but they function as two different entities. The corpus is dominated by bundles of smooth muscle tissue embedded in the ECM. In contrast, the cervix is essentially a fibrous connective tissue organ composed mainly of ECM where collagen and proteoglycans dominate [3,4]. It remains closed despite the increasing pressure of the pregnancy, until final cervical ripening and onset of labor. Ripening of the cervix is a prerequisite for normal labor and delivery during which the non-pliable cervix has to soften and dilate. This implies a dramatic remodeling of the ECM [3]. There are studies on humans showing that cervical ripening shares features with inflammatory reactions [5,6]. A number of neuronal and chemical mediators involved in this process are also known to participate in nociceptive mechanisms. Nerve fibers containing sensory neuropeptides such as substance P (SP) and calcitonin gene related peptide (CGRP) are present in the human and rodent cervix [7-9]. In addition, biomolecules including prostaglandins, nitric oxide (NO) and cytokines are involved in painful inflammatory reactions, nociception and cervical ripening [5,10,11]. The transient receptor potential vanilloid receptor subtype 1 (TRPV1, previously known as VR1) is also a biomolecule associated with inflammatory conditions and nociception. Therefore, we hypothesized that TRPV1 is involved in cervical ripening and nociceptive pathways leading to labor pain. The receptor for the vanilloid capsaicin was first cloned in 1999 [12] and belongs to the family of transient receptor potential (TRP) receptors. TRPV1 is expressed particularly by small-diameter sensory neurons, nociceptors. However, it has also been identified in non-neuronal tissue such as smooth muscle, polymorphonuclear cells and macrophages [13,14]. TRPV1 is a cation channel activated by capsaicin, heat, various lipids and endogenous hydrogen ions released in tissues during inflammation and is regarded as a key molecule in peripheral nociception [15-17]. The aim of this study was to investigate the presence and distribution of TRPV1 in human corpus and cervix uteri during late pregnancy and labor, using immunohistochemical methods and biopsies from non-pregnant subjects as controls.

## Methods

### Patients

Three different groups of patients were studied. One group consisted of eight non-pregnant (NP) women who underwent a hysterectomy because of menorraghia due to myoma. They were all menstruating regularly and none received any hormonal therapy. Biopsies were obtained between cycle day 6 and 24 (six subjects between cycle day 6–14, one on cycle day 24 and two unknown). A second group comprised eight term pregnant (TP) women with normal pregnancy and who had elective caesarean section (CS) prior to onset of labor for one or more of the following reasons: fetal breech position, repeated CS, CS on demand, or vaginal fistula. The third group consisted of eight term pregnant women in established spontaneous labor (TPL). These all had emergency CS due to arrest of labor, fetal malpresentation or fetal distress. Clinical characteristics are given in Table 1. All patients gave their informed, written consent. The Local Ethics Committee of the Karolinska University Hospital approved the study, which was conducted according to the Declaration of Helsinki.

**Table 1:** Clinical characteristics of patients

	**Non-pregnant **n = 8	**Term-pregnant **n = 8	**In labor **n = 8
**Mean age **(years)	43	36	31
Range	39–50	29–42	27–35
**Mean gestational age **Completed weeks		38	40
Range		37–39	39–41
**Mean parity**	2	2	2
Range	1–4	1–4	1–2
**Mean cervical dilatation **(cm)			7
Range			4–10
**Mean birth weight **(g)		3329	4060
Range		2700–4005	3295–5370

### Sampling procedures

Biopsies (400–500 mg) were taken from the isthmic part of the corpus uteri, from now on referred to as corpus. Thus, they were excised from the upper edge of the lower uterine segment where the incision was made during CS. The abdominal serosa and decidua were removed. Biopsies from the corresponding area in the non-pregnant uterus were obtained after hysterectomy. Cervical biopsies (150–300 mg) were taken transvaginally from the anterior cervical lip, including all layers, at the 12 o'clock position, after CS or hysterectomy. All tissues were snap frozen on dry ice and stored at -70°C until use.

### Tissues

Specimens were obtained from the cervix and corpus of the uterus from 3 groups of women: non-pregnant (n = 8), term pregnant before onset of labor (n = 8) and term pregnant in labor (n = 8).

### Antibodies

Primary antibodies used in this study are listed in Table 2. In order to maximize detection of sensory fibers in sections from frozen tissues, the antibodies to the light (peripherin) and heavy subunits of neurofilaments (NFILS) were combined and used as a "cocktail" [18], since peripherin and NFILS antibodies label distinct sub-populations of sensory neurons in dorsal root ganglia.

**Table 2:** Primary Antibodies

**Antibody**	**Host**	**Source:Ref**	**Titer**
**TRPV1**	Rabbit	GlaxoSmithKline, Harlow Essex UK: C22	1:10,000
**Neurofilament Phosphorylated and non-phosphorylated 200 kDa subunit**	Mouse	Dako Cytomation, Ely, UK: Clone N52	1:100,000
**Neurofilament Phosphorylated 200 kDa and 70 kDa subunits**	Mouse	Dako Cytomation, Ely, UK: Clone 2F11	1:10,000
**Peripherin**	Mouse	Novacastra, Newcastle upon Tyne, UK: Clone PJM50	1:500

### Immunohistology

Frozen tissue sections (15 μm) were placed on poly-L-lysine-coated (Sigma, Poole, Dorset, UK) glass slides. The tissue sections were thereafter fixed in 4% w/v paraformaldehyde. Endogenous peroxidase was blocked by incubation in 0.3% w/v hydrogen peroxide in industrial methylated spirit (IMS). After rehydration in phosphate-buffered saline (PBS), sections were incubated overnight with primary antibodies (Table 2). Sites of antibody attachment were revealed using nickel-enhanced, immunoperoxidase method (avidin-biotin complex – ABC elite; Vector Laboratories, High Wycombe, Bucks. U.K.). Negative controls included the omission of primary antibodies or their replacement with non-immune serum. The specificity of TRPV1 antibodies has been described previously [19]. Nuclei were counterstained with 0.1% w/v aqueous neutral red.

For the analysis of NFILS and TRPV1 in the sub-epithelium, positive fibers were counted along the length of the sections in the sub-epithelial area extending 200 microns below the basal lamina. It should be noted that this method will not measure nerve fibers from separate, distinct neurons since fiber profiles may pass in and out of the plane of the sections. Sections were counted by two blinded observers, and results expressed as number of fibers per mm^2^. The intensity of immunostaining of nerve fibers appeared uniform and no attempt was made to measure this since any small variations would be of little value. Since TRPV1-immunoreactive (IR) fibers appeared to be very few throughout the rest of the stroma their frequency was assessed by counting all IR nerve fiber profiles in the entire tissue section (including the sub-epithelial region) from each sample and the total area of the section was measured using a microscope eyepiece graticule. NFILS-IR fibers were much more frequent. They were assessed by counting NFILS-IR nerve fiber profiles in four microscope fields at ×20 objective magnification and these values were compared between groups.

### Statistical analysis

Data were analyzed using Kruskal-Wallis ANOVA by Ranks, Kruskal-Wallis test, p < 0.05 indicated significance.

## Results

### Cervix: TRPV1

**Figure 1:**
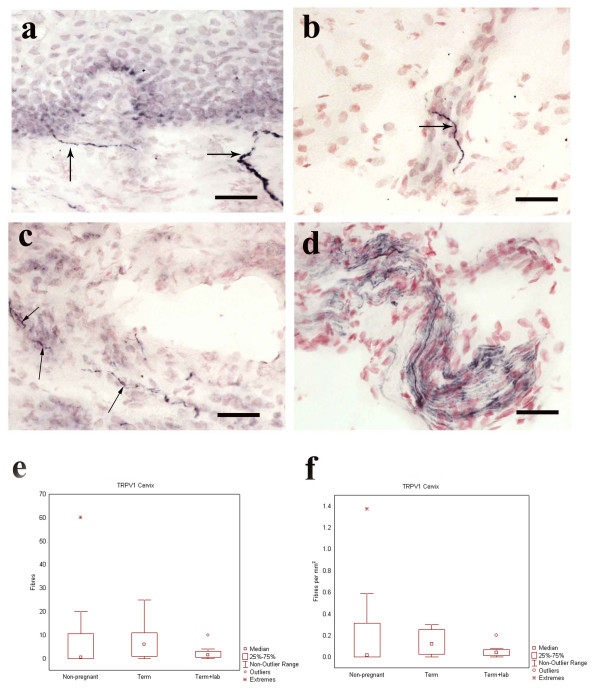
Photomicrographs showing TRPV1-IR nerve fibers in TP cervix (a-c) and a TRPV1-IR nerve fascicle in NP corpus (d). Box plots show the distribution of cervical TRPV1-IR nerve fibers. TRPV1-IR nerve fibers were observed subepithelially (a, arrows) and in the stroma (b, arrow) as well as around blood vessels (c, arrows). Scale bars = 50 μm. TRPV1-IR nerve fibers did not differ significantly between the groups either when presented as total count of positive nerve fibers (e) or as TRPV1 positive nerve fibers/mm^2 ^(f)

Medium to fine caliber TRPV1-IR fibers (Fig. 1a) were detected in all samples, as single fibers or groups of fibers within nerve fascicles scattered throughout the stroma (Fig. 1b) and around blood vessels (Fig. 1c). Comparison of TRPV1-IR nerves between the groups did not show significantly different values (Fig. 1e, 1f).

### Corpus: TRPV1

TRPV1-IR fibers were present within nerve fascicles in controls (Fig. 1d). However, in the TP group, almost all vision fields lacked TRPV1-positive fibers. No TRPV1-IR whatsoever was seen in specimens taken after onset of labor. This very low frequency precluded statistical analysis.

### Cervix: Neurofilament

**Figure 2:**
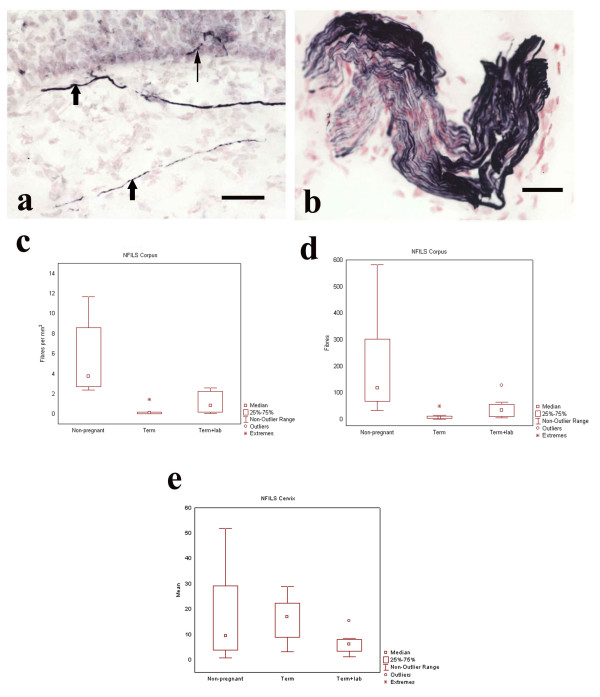
Photomicrographs showing NFILS-IR nerve fibers in TPL cervix (a) and a NFILS-IR nerve fascicle in NP corpus (b). Box plots show the distribution of NFILS-IR nerve fibers in corpus and cervix uteri. NFILS positive fibers were seen in the stroma and in the subepithelial region (a, short arrows). Nerve fibers penetrating the basal epithelium were observed, but only rarely (a, long arrow). A NFILS-IR nerve fascicle is seen in control, non-pregnant corpus (b). Scale bars = 50 μm. The box plot in (c) shows the distribution of NFILS-IR nerve fibers in the three groups of the corpus counted as nerve fibers/mm^2^. There is a significant decrease of IR-nerve fibres/mm^2 ^in the term pregnant group compared to non-pregnant controls, p < 0.0001. A statistically significant decrease of IR-nerve fibers/mm^2 ^is also observed in labor compared to the non-pregnant group, p < 0.01. There is no significant difference between TP and TPL. Comparison of NFILS-IR nerve fibers between the "corpus groups" presented as total count of positive nerves also shows significantly different values (d). There is a statistically significant decrease in the occurrence of IR nerve fibers in the term pregnant compared to the non-pregnant corpus, p < 0.001, and between the non-pregnant group compared to the term pregnant in labor, p < 0.05. However, there is no significant difference between the two pregnant groups. The box plot in (e) shows and compares the distribution of NFILS-IR nerve fibers in the cervical groups. Values did not reach statistical significance.

NFILS-IR fibers were scattered throughout the stroma with only rare fibers penetrating the basal epithelium (Fig. 2a). Counts of IR-nerve fibers in entire sections revealed values which were not significantly different between the groups (Fig. 2e).

### Corpus: Neurofilament

NFILS-IR nerve fibers and fascicles were detected throughout all of the control non-pregnant samples (Fig. 2b), but were much fewer in samples taken at term and labor. There was a significant decrease of NFILS-IR fibers per mm^2 ^in term pregnant specimens compared to controls p < 0.0001, and specimens in labor versus controls p < 0.01 (Fig. 2c). The total count of NFILS-IR nerve fibers also differed significantly (Fig. 2d). There was a significant decrease of total NFILS-IR nerve fibers in term pregnancy compared to the non-pregnant state, p < 0.001 (Fig 2d) and in labor compared to controls, p < 0.05. However, the differences between TP and TPL did not reach significance (Fig. 2c, 2d).

## Discussion

To the best of our knowledge, this study provides the first immunohistochemical evidence for the presence of TRPV1-IR nerve fibers in the human corpus and cervix uteri in the pregnant state and labor. TRPV1-IR fibers disappear almost completely in the pregnant corpus, whilst being retained in the cervix throughout pregnancy and labor.

Changes in uterine innervation are well documented showing a profound decrease of nerve fibers in the pregnant corpus, but a preserved innervation of the cervix [20-22]. The exact reason for this decrease of nerve fibers in the pregnant corpus is not known and still under debate. Both adrenergic and cholinergic nerves appear to be affected [21]. In this study, we used an "NFILS-cocktail" of antibodies as a structural nerve marker in serial sections with TRPV1, since the these antibodies require frozen, post-fixed sections for optimal immunostaining, whereas antibodies to S-100 and the general nerve marker PGP9.5 require immersion fixation for optimal results. However, the results are similar to our previous findings using PGP9.5 antibodies as structural marker [22].

The disparity in innervation of the corpus and the cervix in late pregnancy and labor may possibly reflect the different functions of the corpus and the cervix at the time around parturition.

It is well documented that cervical ripening is an inflammatory process associated with a 10–100 fold increase of cytokines and an influx of cells e.g. leukocytes locally in the cervical tissue [5,6]. Furthermore prostaglandins [23,24], nitric oxide [10], bradykinin [25] are present as well as nerve fibers containing SP and CGRP [7,9]. These biomolecules are also involved in inflammatory induced pain mechanisms. TRPV1 has been documented to be associated with these mediators. Previous studies have shown an up-regulation of cytokines in gastroesophageal reflux disease [26] and an increase of TRPV1-IR nerve fibers in similar diseases such as non-erosive reflux disease and inflamed human oesophagus [27,28]. It is reported that TRPV1 can trigger release of the neuropeptides SP and CGRP from peripheral terminals in inflamed tissues [15]. Bradykinin, an algogenic substance, can increase TRPV1 sensitivity via pathways dependent on protein kinase C or phospholipase C [29-31]. Prostaglandins are suggested to reduce the temperature threshold required for TRPV1 activation [30]. TRPV1 is activated by various ligand-like agents through seemingly unrelated stimuli [30,32]. These observations reveal that TRPV1 is a molecular integrator in inflammatory conditions with a complex multifunctional role [30].

Nerve growth factor (NGF) is another mediator that plays a pivotal role in inflammation and nociception in peripheral tissues. It has a crucial function in the regulation, survival, maintenance and specification of sensory neurons [33,34] and is involved in nociceptive pathways [29,35]. Previous studies have shown a significant increase of TRPV1-IR nerve fibers in inflammatory conditions that elicit pain, such as painful bladder syndrome [18] and vulvodynia [17], and are believed to involve NGF. We have, in an earlier study, demonstrated the occurrence of the NGF receptor p75 in human corpus and cervix uteri in pregnancy and labor [36] and have unpublished data showing mRNA expression for NGFβ and its specific receptor Trk A in biopsies from human corpus and cervix uteri in term pregnancy and labor. Studies in other laboratories have also established links between NGF and TRPV1 where increased TRPV1 levels in peripheral nociceptor terminals are generally restricted to TrkA bearing terminals in inflamed tissue due to NGF mediated activation of p38 MAPK system [13,29,35,37].

## Conclusion

Our results indicate that TRPV1 may be involved in the final remodeling of ECM in the human cervix in pregnancy. This is supported by evidence of interaction with inflammatory mediators. TRPV1-IR nerve fibers persist in the cervix during ripening and labor in contrast to their disappearance in the corpus. This observation suggests that TRPV1, a key molecule in nociception, is involved in the mechanisms underlying labor pain. Our findings indicate the cervix to be an important focus for labor pain. However, further investigations are needed to elucidate the role of TRPV1 in obstetrical pain.

## Competing interests

The author(s) declare that they have no competing interests.

## Authors' contributions

BKT selected and recruited all the patients, collected all the biopsies and participated in evaluating some of the specimens. She also participated in designing the study and drafted the manuscript. PF performed the immunohistochemistry, evaluated the specimens and drafted the manuscript. GEO participated in designing the study, analysis and discussion of the results and critical revision the manuscript. LI participated in designing the study, analysis and discussion of the results and critical revision the manuscript. PA supervised the immunohistochemistry, evaluated the specimens, participated in the analysis and discussion of the results, drafting and critical revision of the manuscript. All authors read and approved the final manuscript.
